# A novel chimeric endolysin Cly2v shows potential in treating streptococci-induced bovine mastitis and systemic infections

**DOI:** 10.3389/fmicb.2024.1482189

**Published:** 2024-10-18

**Authors:** Shuang Wang, Xinxin Li, Junrou Ji, Xiangmin Li, Hechao Zhu, Xiaochao Duan, Dayue Hu, Ping Qian

**Affiliations:** ^1^National Key Laboratory of Agricultural Microbiology, Hubei Hongshan Laboratory, Huazhong Agricultural University, Wuhan, China; ^2^Key Laboratory of Preventive Veterinary Medicine in Hubei Province, the Cooperative Innovation Centre for Sustainable Pig Production, Wuhan, China; ^3^College of Veterinary Medicine, Huazhong Agricultural University, Wuhan, China

**Keywords:** chimeric endolysin, bovine mastitis, therapeutic evaluation, systemic infection, *Streptococcus*, bactericidal activity

## Abstract

*Streptococcus* species are important pathogens implicated in bovine mastitis, causing considerable economic losses within the global dairy industry. With the development of multidrug-resistant bacteria, it is crucial to develop novel antibiotic alternatives. Here, we constructed a novel chimeric endolysin, Cly2v, which comprises the Ply2741 CHAP domain (1-155aa) and the PlyV12 CBD domain (146-314aa). Biochemical characterization analysis indicated that Cly2v exhibits a melting temperature of 50.7°C and retains stable bactericidal activity at pH = 3–10. *In vitro* experiments demonstrated that Cly2v exhibited more efficient bactericidal activity against *Streptococcus* compared to the parental endolysin Ply2741. Cly2v (25 μg/mL) can effectively inhibit and reduce biofilms formed by *Streptococcus*, resulting in a 68 and 44% reduction in OD_590nm_ for *S. agalactiae* X2 and *S. uberis* 002–1 biofilms. Notably, in a mouse mastitis model, treatment with Cly2v (50 μg/gland) led to a reduction in bacterial load by 2.16 log_10_CFU/ml and decreased inflammatory cytokine levels in mammary tissue. To our knowledge, this represents the first application of a chimeric endolysin in the treatment of early-stage mouse mastitis induced by streptococci. Additionally, in a systemic infection model, treatment with Cly2v (400 μg/mouse) provided protection rates of up to 100 and 78% against *S. agalactiae* ATCC13813 infections when challenged for 1 h and 3 h, respectively. Furthermore, a significant reduction in bacterial loads was observed in the blood and organs compared to the PBS group. In summary, Cly2v possesses significant potential as an alternative antibiotic for the treatment of streptococci-induced bovine mastitis and systemic infections.

## 1 Introduction

Bovine mastitis severely threatens the production and quality of the dairy products, resulting in significant substantial economic losses (Kayano et al., 2018; Pang et al., 2018; Guo et al., 2024). As a widespread infectious disease, large-scale dairy farms in seven provinces of China suffered economic losses of up to $76,000 per month due to bovine mastitis between 2015 and 2017 (He et al., 2020). Additionally, mastitis pathogens can be transmitted to humans through unpasteurized milk, posing a threat to public health (Oliver and Murinda, 2012). With the advancement of mastitis diagnostic techniques and the growing awareness of the disease’s impact among livestock veterinarians, bovine mastitis has become a major research highlight in the global veterinary field.

Bovine mastitis represents a multifaceted disease influenced by a range of contributing factors (Viguier et al., 2009). Previous research indicated that approximately 130 pathogens are relevant to bovine mastitis (Kabelitz et al., 2021). Streptococci including *Streptococcus agalactiae*, *Streptococcus uberis*, and *Streptococcus dysgalactiae*, are among the leading pathogens responsible for bovine mastitis worldwide (Ajose et al., 2022; Dyson et al., 2022). Bacteria may spread from infected cows to healthy herds via contaminated milking towels and contact with personnel (Sharun et al., 2021). Additionally, streptococci such as *S. agalactiae* can cause severe infections in humans, posing significant zoonotic risks (Raabe and Shane, 2019). The traditional method for treating and preventing bacterial bovine mastitis relies on antibiotics (Zamojska et al., 2021). However, the increasing prevalence of the antibiotic-resistant bacteria severely limit the effectiveness of these treatments (El-Sayed and Kamel, 2021). Consequently, it is crucial to explore and develop novel alternative agents to treat bovine mastitis caused by streptococci.

Endolysins, which are hydrolytic enzymes derived from bacteriophages, have emerged as a promising novel alternative antimicrobial agent against multi-drug resistant bacteria (Yang et al., 2014b). Unlike phages, endolysins exhibit advantages such as a broad host spectrum, low resistance development, and high safety (Lu et al., 2021). Furthermore, endolysins have exhibited independent bactericidal activity against multidrug-resistant bacteria. Fischetti et al. found that PlySs2 exhibits broad bactericidal activity against *Streptococcus*, *Staphylococcus*, and *Enterococcus* species, making it the lysin with the broadest known bactericidal spectrum (Gilmer et al., 2013). Doehn et al. found that Cpl-1 not only prevents acute otitis media in mouse models but also treats systemic infections caused by *Streptococcus pneumoniae* (McCullers et al., 2007; Doehn et al., 2013). Additionally, endolysins generally have a modular structure, which allows for the replacement and modification, providing more options for clinical treatment (Murray et al., 2021). A few studies have demonstrated that chimeric endolysins possess broader potential for application compared to their parental endolysins. Yang et al. developed a chimeric lysin ClyH, which exhibited remarkable bactericidal activity against Methicillin-resistant *Staphylococcus aureus* (MRSA) strains compared to lysostaphin and its parental endolysin (Yang et al., 2014b). Duan et al. demonstrated that, compared to LysGH15, the chimeric endolysin ClyQ not only has a broader lytic spectrum but also diminished the onset of bacterial resistance when used in combination with mupirocin (Duan et al., 2023). However, to our knowledge, there are few reports on chimeric endolysins aimed at treating bovine mastitis induced by streptococci.

Here, we constructed a novel chimeric lysin, Cly2v, which consists of the Ply2741 CHAP domain and the PlyV12 cell-wall binding domain (CBD). Compared to its parental endolysin Ply2741, Cly2v exhibited more efficient bactericidal activity against *Streptococcus* species causing bovine mastitis and significantly inhibited and removed bacterial biofilms. In addition, in a mouse mastitis model, Cly2v significantly reduced bacterial load and inflammatory cytokine levels in mammary tissue. Meanwhile, Cly2v could treat systemic infections caused by *S. agalactiae* ATCC13813. Our study not only provides a novel antibiotic alternative to combat streptococci-induced bovine mastitis and systemic infections but also enhances the understanding of the engineering of novel chimeric endolysins.

## 2 Materials and methods

### 2.1 Cells and bacteria

HEK-293 T, PK15, and IPEC-J2 cells were cultured in DMEM medium (Gibco, United States), supplemented with 10% (v/v) fetal bovine serum (FBS) (Sigma-Aldrich, United States), and incubated at 37°C in a 5% CO_2_ atmosphere. *Streptococcus* were cultured in Tryptic Soy Broth (TSB) and Tryptic Soy Agar (TSA) media (BD, United States) containing 5% (v/v) FBS (Every Green, China). The strains used in this research are detailed in Supplementary Table S1.

### 2.2 Construction, expression, and purification of Cly2v

The genomic fragments encoding the endolysins PlyV12 (Genbank ID: AAT01859.1) and Ply2741 (Genbank ID: PQ213358) were synthesized by Tsingke Biotechnology, Co., Ltd. The chimeric endolysin Cly2v was constructed by fusing the Ply2741 CHAP domain (1-155aa) with the PlyV12 CBD domain (146-314aa) via overlap PCR. The Cly2v gene was subsequently cloned into the pCold™ II plasmid (Takara, Japan) following digestion with *BamH*I and *Hind*III. The specific primers used in this research are detailed in Supplementary Table S2.

The recombinant plasmid pCold-Cly2v was introduced into *E. coli* BL21 (DE3) competent cells and grown in LB medium supplemented with 100 μg/mL ampicillin at 37°C until OD_600nm_ reached to 0.6. After inducing with 0.6 mM Isopropyl *β*-D-thiogalactoside (IPTG) at 16°C for 18 h, the cells were harvested by ultracentrifugation and resuspended in binding buffer (20 mM Tris, 150 mM NaCl, pH = 7.5). The samples were broken using a cell pressure crusher and centrifuged at 10000 × g for 20 min at 4°C to harvested the crude recombinant protein. And then, the crude endolysin was purified via affinity chromatography (AFC) and size exclusion chromatography (SEC) according to the protocols. Briefly, His-tagged protein was purified using a His-Trap FF column (Cytiva, United States) with elution buffer (20 mM Tris, 150 mM NaCl, 300 mM imidazole pH = 7.5), followed by further purification using a HiLoad Superdex 200pg column (Cytiva, United States) with SEC buffer (20 mM Tris, 150 mM NaCl, pH = 7.5).

### 2.3 Bioinformatics and structural analysis of Cly2v

The physicochemical properties and hydrophobicity of Cly2v were analyzed using Expasy.^1^ The three-dimensional (3D) structure prediction was conducted via the Colabfold webserver (Mirdita et al., 2022), with visualization and analysis performed using PyMOL. The secondary structure was further determined through circular dichroism as described previously (Luo et al., 2020). Briefly, the endolysin was adjusted to a concentration of 0.5 mg/mL in SEC buffer. The experiments were conducted on a J-1500 CD Spectrometer using a 0.1 cm quartz cuvette (JASCO, Japan), measuring wavelengths from 190 nm to 260 nm. The CDNN V2.1 software was used to calculate the secondary structure results.

### 2.4 Biological characterization of Cly2v

The effect of temperature and pH on bactericidal activity were determined by turbidity reduction assay as described previously (Gilmer et al., 2013). Briefly, Cly2v was incubated at different temperatures. Then, 100 μL of Cly2v were taken every 10 min and mixed with 100 μL of *S. agalactiae* ATCC13813, which adjusted with PBS to OD_600nm_ = 0.8 (approximately 2.5 × 10^8^ CFU/mL). After incubation at 37°C for 30 min, the absorbance at OD_600nm_ was determined, with PBS serving as the negative control. For the pH stability, Cly2v was mixed with different pH buffers (20 mM sodium acetate buffer (pH 3.0 to 6.0), 20 mM sodium phosphate buffer (pH 7 to 8), and 20 mM Tris–HCl buffer (pH 9.0 to 10.0)) and incubated at 37°C for 30 min. To ensure consistent pH conditions, the bacteria were also resuspended in the respective pH buffers and adjusted to OD_600nm_ = 0.8 (approximately 2.5 × 10^8^ CFU/mL). Subsequently, Cly2v was mixed with the bacteria and incubated at 37°C for 30 min before measuring the absorbance at OD_600nm_. Different pH buffers served as controls to reduce experimental errors from pH effects on bacterial viability. The relative activity of Cly2v under different temperature and pH conditions was expressed as a percentage of the maximum enzyme activity. All experiments were conducted in triplicate.

The thermal stability of Cly2v was assessed using nano differential scanning fluorimetry (nanoDSF) with the Prometheus NT.48 (Nano Temper, Germany). The experiments were conducted over a temperature range of 20°C to 95°C, with a rate of 1°C/min. The first derivative F350/F330 was automatically calculated and the melting temperature (Tm) was determined at the peak of the derivative curve.

### 2.5 Lytic spectrum and bactericidal activity assay

To explore the lytic spectrum of Cly2v against *Streptococcus* species that caused mastitis, the turbidity reduction assay was conducted as described previously (Gilmer et al., 2013; Li et al., 2023). Mid-logarithmic phase bacteria were adjusted with PBS to OD_600nm_ = 0.6–0.8. Then, 100 μL of the bacteria was mixed with 100 μL of endolysin (50 μg/mL) in a 96-well plate. The absorbance was measured at OD_600nm_ after a 30-min incubation at 37°C. The turbidity reduction ratio was calculated as described below. Ply2741 was used as the parental endolysin control.

To evaluate the bactericidal efficiency of Cly2v, logarithmic phase bacteria were mixed with Cly2v (100 μg/mL), while PBS served as a negative control. Following a 1 h incubation at 37°C, the mixture was plated on TSA to determine bacterial counts. Moreover, the bactericidal efficiency of Cly2v was evaluated via bactericidal kinetics curve assay. In brief, Cly2v (50 μg/mL) was incubated with bacteria, and bacterial counts were determined by serial dilutions on TSA plates at 5 min intervals for 45 min.


$$\mathrm{Decrease}\;\mathrm{of}\;\mathrm{turbidity}\left(\%\right)=\frac{OD600\mathrm{nm}\left(PBS\right)-OD600\mathrm{nm}\left(\mathrm{endolysin}\right)}{OD600\mathrm{nm}\left(PBS\right)}$$

### 2.6 Evaluation of biofilm formation ability in *Streptococcus*

The biofilm-formation ability of *Streptococcus* was determined via the crystal violet staining method as described previously (Wang et al., 2023). Briefly, overnight cultured bacteria (~5 × 10^8^ CFU/mL) were diluted in TSB medium at a ratio of 1:100. Subsequently, 100 μL of the bacteria and 100 μL of PBS were inoculated into a 96-well plate to form biofilms, while TSB medium served as the control. After incubation at 30°C for 24 h, the wells were washed three times with PBS and dried at room temperature. Each well was stained with 200 μL of crystal violet and incubated at 37°C for 30 min. Finally, after washing the wells with PBS, 33% acetic acid was added to each well to dissolve the stain, and absorbance was measured at 590 nm. The experiments were conducted for three times.

### 2.7 Antibiofilm activity of Cly2v

To investigate the inhibitory ability of Cly2v against biofilms, overnight cultures of *S. agalactiae* X2 and *S. uberis* 002–1 (~5 × 10^8^ CFU/mL) were diluted at a 1:100 ratio. Subsequently, the bacteria were mixed with different doses of Cly2v (5 μg/mL, 10 μg/mL, and 25 μg/mL) and ampicillin (5 μg/mL) in a 96-well plate to form biofilms after incubation at 30°C for 24 h, with PBS serving as the control. Some wells were washed three times with PBS, resuspended in PBS, and then plated on TSA plates for colony counting. In other wells, biofilm formation ability was determined using the crystal violet staining method.

To investigate the effect of Cly2v on mature biofilms, *S. agalactiae X2* and *S. uberis* 002–1 (~5 × 10^8^ CFU/mL) were diluted at a 1:100 ratio and inoculated into a 96-well plate without treatment to form biofilms. After incubation at 30°C for 24 h, different doses of Cly2v (10 μg/mL, 25 μg/mL, and 50 μg/mL) and ampicillin (5 μg/mL) were added to the wells, and the mixture was incubated at 37°C for 3 h. After incubation, the biofilms were determined by crystal-violet staining method and bacterial counts as described above. All experiments were conducted in triplicate.

### 2.8 Scanning electron microscope

The morphological changes of bacterial mature biofilms treated with Cly2v were observed using scanning electron microscopy (SEM) as described previously (Duan et al., 2023). Briefly, mid-logarithmic phase bacteria (~2 × 10^8^ CFU/mL) were inoculated at a 1:100 ratio in a 24-well plate with coverslips and cultured at 30°C for 24 h to form biofilms. Subsequently, Cly2v (100 μg/mL) was added and the mixture were incubated at 37°C for 3 h to remove the biofilms. The samples were then washed with PBS and fixed with 2% glutaraldehyde for 2 h, followed by six washes with ddH_2_O. Finally, after spraying with gold, the coverslips were observed using scanning electron microscope (NTC JSM-6390LV, Japan).

### 2.9 Bactericidal activity of Cly2v in milk

The bactericidal activity of Cly2v in milk was evaluated as described previously (Wang et al., 2023). In brief, *S. agalactiae* ATCC13813 (1 × 10^5^ CFU/mL) was grown in the whole milk at 37°C. After incubation for either 1 h and 2 h, 50 μg/mL of Cly2v was added to the milk and incubated at 37°C for 1 h. Subsequently, samples were serially diluted in ten-fold and plated on the TSA plates for colony counting. All experiments were conducted in triplicate.

### 2.10 Toxicity of Cly2v on mammals’ cells and mouse tissues

To evaluate the toxicity of Cly2v on mammalian cells, a CCK-8 assay was carried out as described previously (Zhao et al., 2018). Cells, specifically PK15, IPEC-J2, and HEK-293 T were seeded at a density of 1 × 10^5^ cells per well. Subsequently, cells were exposed with varying concentrations of Cly2v (200 μg/mL and 400 μg/mL) and incubation for 2 h, with PBS serving as the control. Cells viability was evaluated via the CCK-8 kit (MCE, United States) following the protocols, and the absorbance was measured at 450 nm. All the experiments were conducted for three times.

The toxicity of Cly2v to mouse tissues was further evaluated in a mouse model. Female specific-pathogen-free (SPF) BALB/c mice were divided into 3 groups (*n *= 5) and each group received an intraperitoneal injection of 400 μg or 800 μg of Cly2v, with PBS serving as the control. The survival rate and physiological condition of the mice were monitored for 5 days, and mouse organs were collected for pathological analysis at 5 d.

### 2.11 Mouse mastitis model

A mouse mastitis model was constructed as described previously to explore the efficacy of Cly2v against the bovine mastitis *in vivo* (Gutiérrez et al., 2020; Li et al., 2023). Briefly, female SPF BALB/c mice were synchronized for pregnancy by circadian cycle adjustment. At 14 to 21 days postpartum, the pups were allowed to nurse for 1 h to fully deplete the milk. Subsequently, the offspring were removed to establish the mastitis model while ensuring that the lactating females remained in the lactation period. Mice were anesthetized with 1.25% avertin, and a small incision of approximately 0.5 mm was made on the L4 and R4 mammary glands to expose the mammary duct. Then, *S. agalactiae* ATCC13813 (10^4^ CFU/gland) was administered through the mammary duct, except for the control group. After 1 h post-infection, the L4 and R4 mammary glands were injected with PBS, Ply2741 (50 μg/gland), Cly2v (50 μg/gland), or Ceftiofur (25 μg/gland) to establish the PBS-treated group (*n* = 5), Ply2741-treated group (*n* = 5), Cly2v-treated group (*n* = 5), and antibiotic-treated group (*n* = 5). At 48 h post-treatment, the mice were humanely euthanized, and the L4 and R4 mammary glands were harvested and mixed. The mammary tissue samples were completely homogenized and serially diluted in a ten-fold series for viable cell counting. Inflammatory cytokine levels (TNF-*α* and IL-6) in the mammary tissues were determined via qRT-PCR assay. Additionally, mammary tissues were collected for histopathological analysis after 48 h of treatment.

### 2.12 RNA extraction, reverse transcription, and qRT-PCR

The mRNA expression levels of inflammatory cytokine (TNF-α and IL-6) were determined by qRT-PCR assay (Wang et al., 2023). Briefly, TRIpure reagent (Aidlab, China) was used to extracted total RNA from mammary tissues. Subsequently, the extracted RNA was then reverse transcribed into cDNA, and qRT-PCR assay was performed on Applied Biosystems ViiA™7 system (Thermo Fisher Scientific, United States) with Hieff ® qPCR SYBR Green Master Mix (Yeasen, China). The formula 2^-ΔΔCt^ was used to analyzed the data and the GAPDH gene was served as the internal gene. The primers used in this assay are detailed in Supplementary Table S2.

### 2.13 Mouse systemic infection model

Five-week-old female SPF mice were randomly divided into 6 groups (*n* = 6 for each group) and each group injected intraperitoneally with *S. agalactiae* ATCC13813 at doses of 4 × 10^9^ CFU, 2 × 10^9^ CFU, 1 × 10^9^ CFU, 5 × 10^8^ CFU, and 2.5 × 10^8^ CFU, respectively. Equal volumes of PBS were served as a control. The survival rate of mice was monitored and recorded for 48 h to determine the lethal dose. The minimum challenge dose that resulted in the death of all mice within 48 h was used for the survival rate experiment, while the maximum challenge dose that all mice survived was used for the bacterial load assay.

To explore the therapeutic effect of Cly2v on systemic infection caused by *S. agalactiae*, mice were randomly divided into 4 groups (*n* = 6 per group), Group I, II, III, and IV. Group I, II, and III was injected intraperitoneally with 1 × 10^9^ CFU of *S. agalactiae* ATCC13813, with Group IV received an equal volume of PBS serving as the control. After 1 h post-infection, group II, III, and IV were treated with 200 μg or 400 μg doses of Cly2v, respectively, while the Group I received PBS as the control. The survival rate of mice was monitored for 5 d. Additionally, a survival experiment was established for treatment with Cly2v at 3 h post-infection. Mice were randomly divided into 3 groups (*n* = 9 per group) and were intraperitoneally injected with 1 × 10^9^ CFU of *S. agalactiae* ATCC13813. After 3 h of infection, the mice in each group were treated with 200 μg or 400 μg of Cly2v, with an equal volume of PBS serving as the control. The survival rate of the mice was recorded for 5 days.

For bacterial load assay, mice were randomly divided into 2 groups (*n *= 9 per group), and each mouse was injected intraperitoneally of 2.5 × 10^8^ CFU of *S. agalactiae* ATCC13813. The treatment group received 200 μg of Cly2v, while the control group was injected with PBS at 1 h post-infection. Mice were humanely euthanized after 6, 18, and 30 h of treatment, and blood, heart, lungs, liver, spleen, and kidneys organs were harvested. After homogenization and ten-fold dilution, the samples were plated on TSA plates for colony counting to determine the bacterial load in the organs.

### 2.14 Statistical analysis

Data are presented as mean ± standard deviation (SD) and the normality of the data was analyzed using the Anderson-Darling test (*N* > 6) and Quantile-Quantile plots (*N* < 6). Statistical significance was analyzed using one- and two-way analysis of variance (ANOVA) with GraphPad Prism (version 8). *p values* < 0.05 was considered statistically significant (*).

## 3 Results

### 3.1 Construction and structure analysis of Cly2v

**Figure 1:**
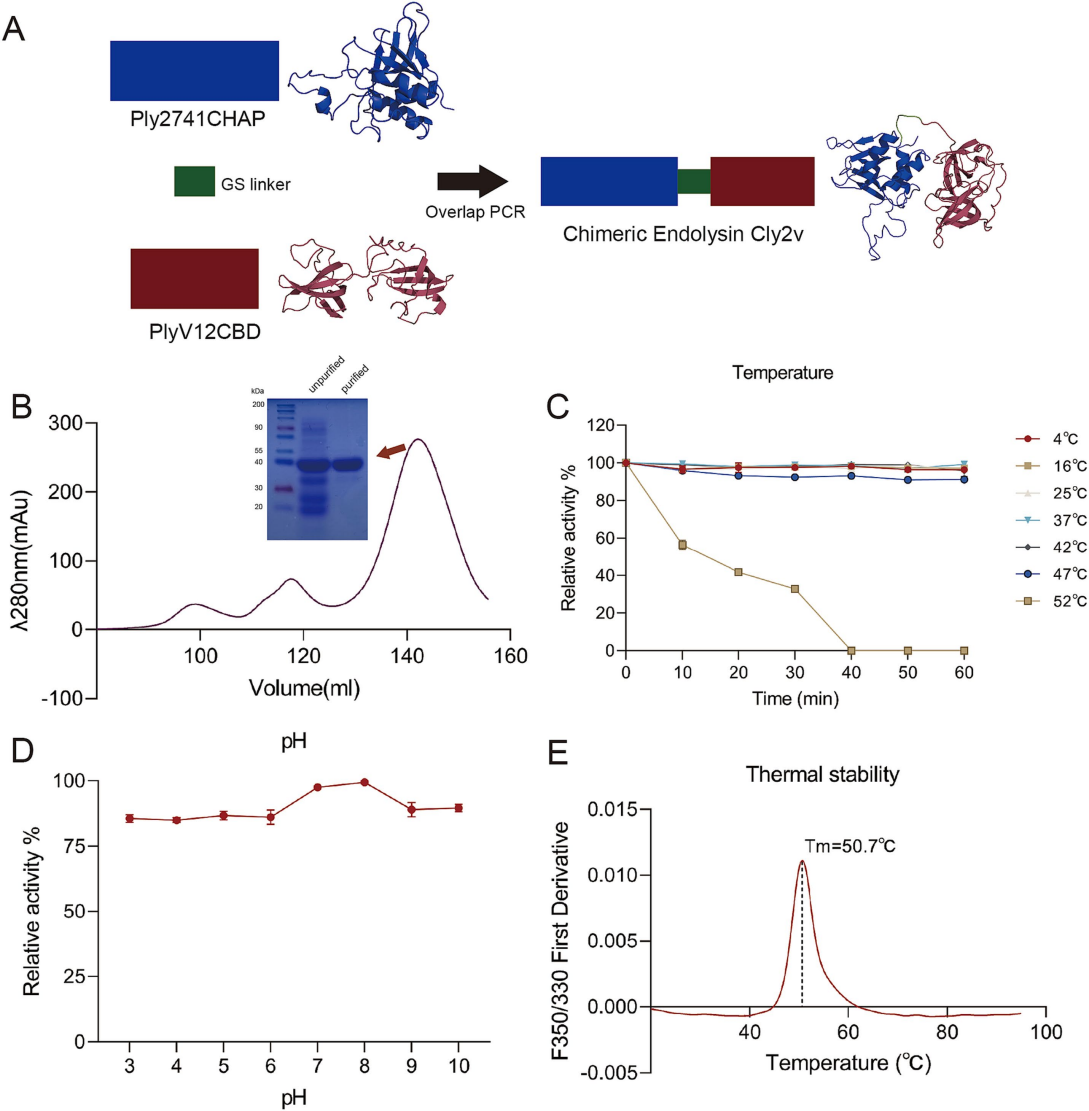
Construction and structure analysis of the chimeric endolysin Cly2v. **(A)** Schematic diagram and 3D structure of Cly2v. Cly2v was composed of the CHAP domain from Ply2741 and the CBD domain from PlyV12. The 3D structure was predicted using ColabFold. **(B)** Cly2v was purified by size exclusion chromatography and identified by SDS-PAGE gel, with the Cly2v protein having a molecular weight of 36 kDa. **(C,D)** Bactericidal activity of Cly2v under different temperatures **(C)** and pH conditions **(D)**. Results are the means of three independent experiments. **(E)** The thermal stability analysis of Cly2v, with the spectra determined from 20°C to 95°C. Results represent the means of three independent experiments.

To enhance the bactericidal activity of endolysins, we constructed a novel chimeric endolysin, Cly2v. Cly2v consisted of the CHAP domain from the *S. suis* endolysin Ply2741 and the CBD domain from the *E. faecalis* phage endolysin PlyV12. These two domains were connected by a flexible linker (GGSSGS) (Figure 1A). As shown in Supplementary Figure S1A, physicochemical analysis indicated that the theoretical isoelectric point and instability index of Cly2v were 9.52 and 27.69, respectively. Hydropathy analysis revealed that the GRAVY index of Cly2v was −0.413, with a higher number of hydrophilic residues compared to hydrophobic residues, indicating that Cly2v had the potential for soluble expression *in vitro*. After purification by AFC and SEC, Cly2v exhibited high purity in SDS-PAGE gel with the expected molecular weight (Figure 1B).

Additionally, circular dichroism and colabfold were used to analyze the secondary structure of Cly2v. The results showed that the proportions of *α*-helices, *β*-strands, and coils in Cly2v were 6.7, 41.9, and 33.4%, respectively (Supplementary Figure S1B). Specifically, the Cly2v consisted of 5 α-helices and 15 β-strands (Supplementary Figure S1C).

### 3.2 Biochemical characterization of Cly2v

The influence of temperature and pH on the bactericidal activity of the chimeric endolysin Cly2v were further evaluated. The results showed that Cly2v maintained over 90% activity between 4°C and 47°C. However, the bactericidal activity decreased significantly when the temperature increased to 52°C, and Cly2v was completely inactivated after incubation for 40 min at 52°C (Figure 1C). As shown in Figure 1D, Cly2v was able to maintain the bactericidal activity between pH = 3.0–10.0, with an optimal pH of 7.0–8.0. Additionally, thermal stability analysis revealed that the melting temperature (Tm) of Cly2v was 50.7°C, indicating that Cly2v could undergo denaturation and unfolding at temperatures above 50.7°C (Figure 1E). This result was consistent with the observation that Cly2v was inactivated at 52°C.

### 3.3 Cly2v exhibits broad-spectrum and high-efficiency bactericidal activity against streptococcal species causing mastitis *in vitro*

**Figure 2:**
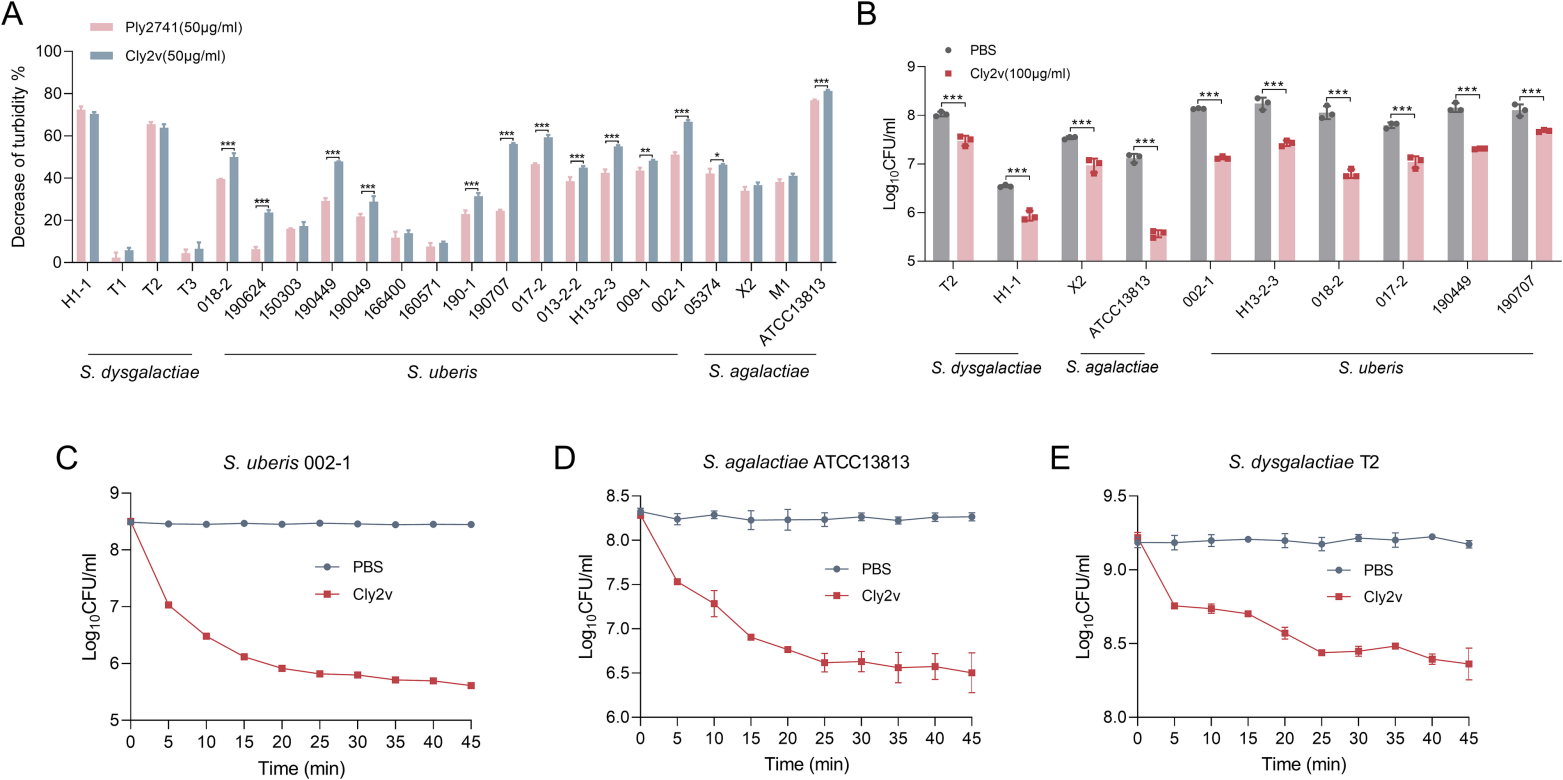
Host spectrum and bactericidal activity of Cly2v. **(A)** Host spectrum of Cly2v and parental endolysin Ply2741. Different *Streptococcus* (*S. agalactiae*, *S. dysgalactiae*, and *S. uberis*) were used as test strains. **(B)** Bactericidal activity of Cly2v against various *Streptococcus*. The number of viable bacteria was evaluated after treatment with 100 μg/mL of Cly2v for 1 h. **(C–E)** Bactericidal kinetics curve of Cly2v against *Streptococcus*. The number of bacteria was measured over 45 min following the mixing of 50 μg/mL of Cly2v with the *S. uberis* 002-1 **(C)**, *S. agalactiae* ATCC13813 **(D)**, and *S. dysgalactiae* T2 **(E)**. Experimental data are shown as the mean of three independent experiments, with statistical analysis performed using two-way ANOVA (*, *p* < 0.05; **, *p* < 0.01; ***, *p* < 0.001).

As shown in Figure 2A, Cly2v exhibited broad-spectrum bactericidal activity against various strains, including *S. agalactiae*, *S. dysgalactiae*, and *S. uberis*. Specifically, Cly2v demonstrated lytic efficiency greater than 40% in 12 out of 22 tested strains. More importantly, Cly2v exhibited higher bactericidal activity against streptococcal species causing mastitis compared with the parental endolysin Ply2741, indicating that Cly2v, a chimeric endolysin constructed based on the Ply2741, had better potential for the treatment of streptococci-induced mastitis in dairy cows.

Additionally, the bactericidal activity of Cly2v was determined by measuring changes in bacterial colony counts after treatment. The results showed that Cly2v significantly reduced the bacterial count in all 10 selected streptococcal strains, particularly in *S. agalactiae* ATCC13813 strain, where 100 μg/mL of Cly2v led to a reduction from 7.12 log_10_CFU/ml to 5.57 log_10_CFU/ml, resulting in a reduction of 97.22% in bacterial count (Figure 2B). Subsequently, we evaluated the bactericidal kinetics curve of Cly2v. The results showed that 50 μg/mL of Cly2v was able to reduce the bacterial count of *S. uberis* 002–1 from 8.47 log_10_CFU/ml to 5.82 log_10_CFU/ml (a reduction percentage of 99.77%) (Figure 2C), *S. agalactiae* ATCC13813 from 8.24 log_10_CFU/ml to 6.63 log_10_CFU/ml (a reduction percentage of 97.56%) (Figure 2D), and *S. dysgalactiae* T2 from 9.18 log_10_CFU/ml to 8.44 log_10_CFU/ml (a reduction percentage of 81.70%) (Figure 2E) within 25 min.

### 3.4 Cly2v significantly inhibits and reduces biofilms of *Streptococcus*

**Figure 3:**
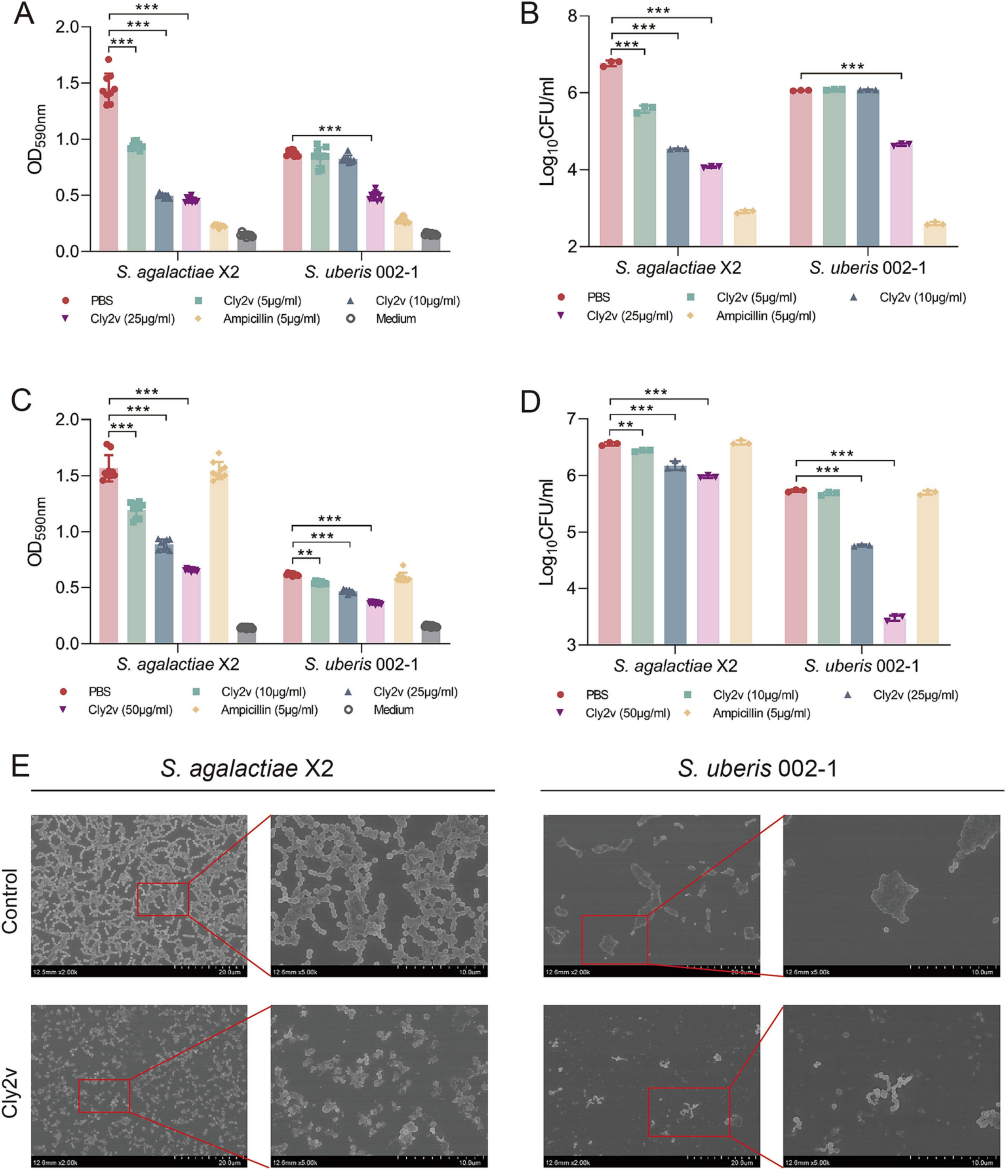
Effects of Cly2v on bacterial biofilms. **(A,B)** The inhibitory effect of Cly2v on biofilm formation was measured by absorbance at OD_590nm_ **(A)** and viable cell counts **(B)** after mixing different dose of Cly2v with bacteria. **(C,D)** Effect of Cly2v on mature bacterial biofilms. The absorbance at OD_590nm_ **(C)** and the number of viable cells **(D)** were measured after treatment with different dose of Cly2v. The experimental results are presented as the mean of three independent experiments. Statistical significance was analyzed using two-way ANOVA (* *p* < 0.05; ** *p* < 0.01; *** *p* < 0.001). **(E)** SEM results of Cly2v (100 μg/mL) removing mature biofilms of *S. agalactiae* X2 and *S. uberis 002–1*. Magnification: × 2000 (left) and × 5,000 (right).

Among the 18 tested strains, *S. agalactiae* X2, *S. uberis* 002–1, *S.dysgalactiae* T1, and *S. uberis* 018–2 showed the ability to form strong biofilms (Supplementary Figure S2). Due to the poor lytic activity of Cly2v against 018–2 and T1, we selected the strains *S. agalactiae* X2 and *S. uberis* 002–1 for subsequent experiments. The results showed that Cly2v significantly inhibited biofilm formation in both *S. agalactiae* X2 and *S. uberis* 002–1. At a concentration of 25 μg/mL, Cly2v decreased the OD_590nm_ absorbance of biofilms by 68% (*p* < 0.001) and 44% (*p* < 0.001) for X2 and 002–1 (Figure 3A), respectively, while the bacterial counts decreased from 6.77 log_10_CFU/ml to 4.07log_10_CFU/ml and from 6.06 log_10_CFU/ml to 4.65 log_10_CFU/ml, corresponding to reductions of 99.80 and 96.09% (Figure 3B). Compared to Cly2v, ampicillin exhibited a stronger inhibitory effect on biofilm formation, with OD_590nm_ absorbance decreasing by 84 and 68%, respectively. For established biofilms, Cly2v can significantly remove the biofilms in a dose-dependent manner. As shown in Figure 3C, Cly2v (50 μg/mL) significantly reduced the OD_590nm_ absorbance of biofilms by 58% for X2 (*p* < 0.001) and 42% for 002–1 (*p* < 0.001). Additionally, Cly2v decreased the viable cell counts from 6.56 log_10_CFU/ml to 5.98 log_10_CFU/ml for X2 and from 5.73 log_10_CFU/ml to 3.48 log_10_CFU/ml for 002–1, corresponding to reductions of 73.74 and 99.45% (Figure 3D). Notably, ampicillin demonstrated no effect on mature biofilms.

To observe the morphological changes in bacterial biofilms after Cly2v treatment, we employed scanning electron microscopy (SEM) experiment. The results demonstrated that, compared to the control group, the established biofilms treated with Cly2v exhibited a loose adherence, with no contiguous biofilm formation and a significant reduction in viable cell counts (Figure 3E). These results indicated that Cly2v not only significantly inhibited the formation of bacterial biofilms but also effectively removed mature biofilms.

### 3.5 Cly2v effectively reduces streptococci in whole milk

Streptococci-induced bovine mastitis can adversely impact milk quality. Therefore, we investigated the bactericidal activity of Cly2v against *S. agalactiae* ATCC13813 in whole milk. As shown in Supplementary Figure S3, after incubating milk with *S. agalactiae* ATCC13813 for 1 h, the addition of Cly2v led to a significant decrease in bacterial counts, with the counts decreasing from 6.51 log_10_CFU/ml to 6.09 log_10_CFU/ml, corresponding to a reduction of approximately 62.0% (*p* < 0.05).Additionally, Cly2v was added to infected milk at 100 μg/mL after 2 h infection, and the bacterial count decreased from 7.15 log_10_CFU/ml to 6.49 log_10_CFU/ml (approximately 74.2% reduction, *p* < 0.01). These results indicated that Cly2v could significantly reduce streptococci in whole milk.

### 3.6 Cly2v exhibits significant therapeutic potential against early-stage streptococci-induced mastitis in mice

**Figure 4:**
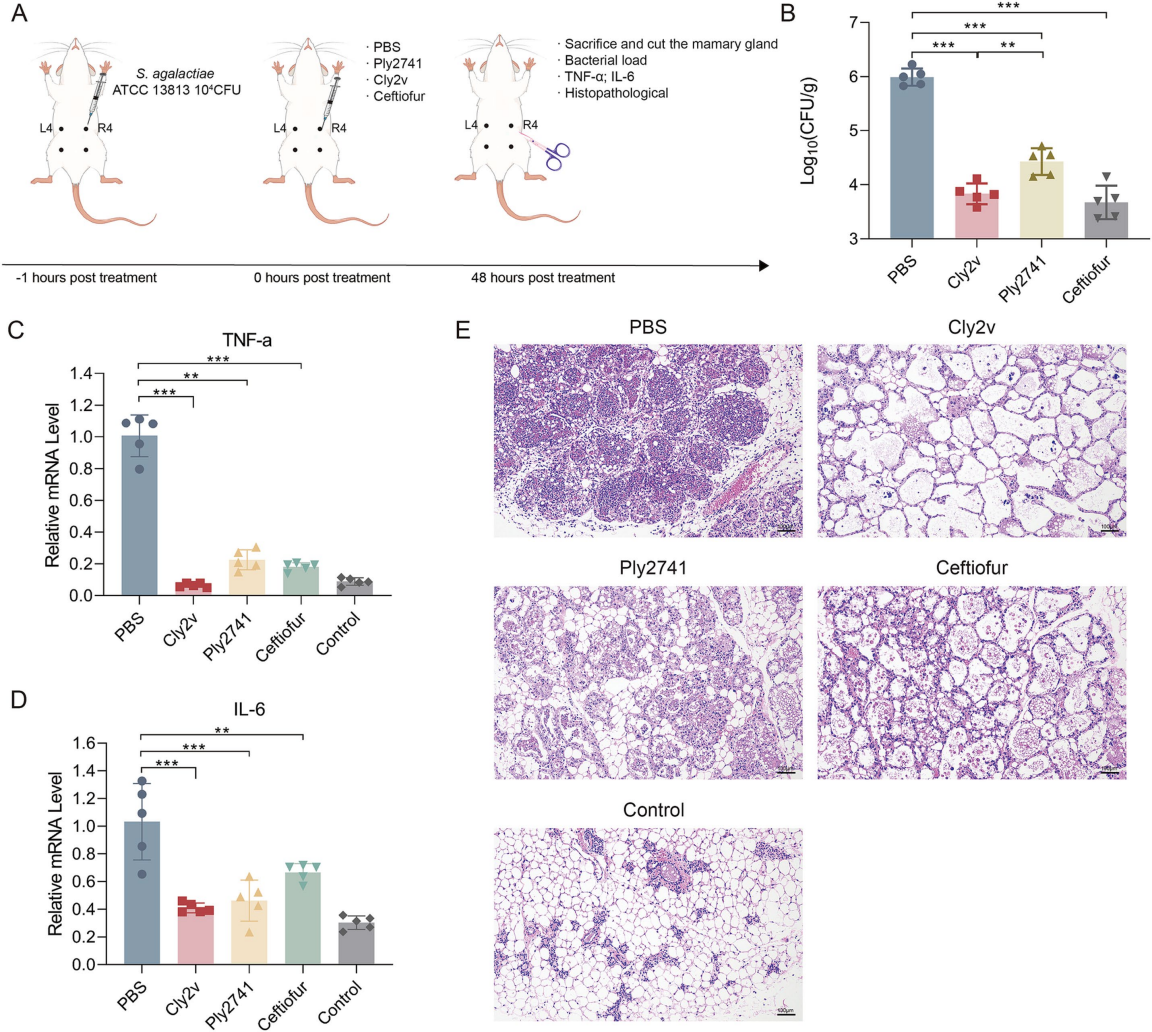
Therapeutic effects of Cly2v on early-stage *S. agalactiae* ATCC13813-induced mouse mastitis. **(A)** Schematic representation of the early-stage mouse mastitis treatment model. **(B)** Bacterial load of *S. agalactiae* ATCC13813 in mouse mammary tissue after treatment with Cly2v (50 μg/gland), Ply2741 (50 μg/gland), and ceftiofur (25 μg/gland). **(C,D)** The mRNA levels of TNF-*α* **(C)** and IL-6 **(D)** in mouse mammary tissue measured by qRT-PCR and analyzed by the formula 2^-ΔΔCt^ after treatment. **(E)** Histopathological changes in mouse mammary tissue after treatment with PBS, Cly2v, Ply2741, ceftiofur, and control. The experimental results are presented as the mean of three independent experiments. Statistical significance was analyzed using one-way ANOVA (* *p* < 0.05; ** *p* < 0.01; *** *p* < 0.001).

To investigate the therapeutic effect of Cly2v in bovine mastitis, we established a mouse mastitis model and treated it with Cly2v. Ply2741 and ceftiofur were served as the parental endolysin control and positive control, respectively (Figure 4A). As shown in Figure 4B, Cly2v significantly reduced the bacterial burden in mouse mammary tissues, decreasing the bacterial counts from 5.99 log_10_CFU/g to 3.83 log_10_CFU/g, representing a reduction of 99.3% (*p* < 0.001). Additionally, compared to the parental endolysin Ply2741, the reduction in bacterial burden in mouse mammary tissues after Cly2v treatment was more substantial (*p* < 0.01). Notably, the ceftiofur-group also reduced bacterial counts to 3.68 log_10_CFU/g, comparable to the effect observed with Cly2v treatment (*p* > 0.05). Moreover, Cly2v, Ply2741, and ceftiofur treatments significantly decreased the mRNA levels of inflammatory factors in mouse mammary tissues. After treatment with Cly2v, TNF-*α* and IL-6 levels decreased by 15.77-fold (Figure 4C) and 2.51-fold (Figure 4D), respectively, with no significant difference compared to the antibiotic group. Histopathological analysis revealed that after infection with *S. agalactiae*, there was significant thickening of the alveolar wall in the mammary tissue of mice, accompanied by severe edema, congestion, and a large number of inflammatory cells and erythrocyte infiltration, compared to the control group. After treatment with Cly2v, Ply2741, and ceftiofur, the mammary tissues exhibited minimal pathological alterations, with the Cly2v-group showing the least pathological damage (Figure 4E). These results indicated that Cly2v significantly reduced bacterial load in early-stage mouse mastitis models, demonstrating its potential as a treatment for streptococci-induced mastitis.

### 3.7 Cly2v exhibits no significant toxicity in mammalian cells and mice organs

To evaluate the toxicity of Cly2v on mammalian cells, a CCK8 assay was employed. As shown in Supplementary Figure S4A, the results showed that the survival rates of HEK-293 T, PK15, and IPEC-J2 cells exceeded 100% at concentrations at 200 μg/mL and 400 μg/mL, indicating that Cly2v exhibited no significant toxicity to mammalian cells. Additionally, the toxicity evaluation results indicated that mice receiving intraperitoneal injections of 400 μg, and 800 μg of Cly2v exhibited a 100% survival rate and maintained normal physiological conditions (Supplementary Figure S4B). Pathological analysis indicated that, similar to the PBS group, mice administered 800 μg of Cly2v exhibited no significant pathological damage in their organs (Supplementary Figure S4C).

### 3.8 Cly2v exhibits therapeutic efficacy in mice with systemic streptococci infections

To investigate the therapeutic effects of Cly2v on systemic infection caused by *Streptococcus*, we first determined the optimal challenge dose of *S. agalactiae* ATCC13813. The mice challenged with a dose of 1 × 10^9^ CFU/mouse died within 48 h, while those infected with a dose of 2.5 × 10^8^ CFU survived for 48 h (Supplementary Table S3). Consequently, the optimal infection dose of *S. agalactiae* ATCC13813 for the survival rate experiment was 1 × 10^9^ CFU, while the challenge dose for the bacterial load assay was 2.5 × 10^8^ CFU.

**Figure 5:**
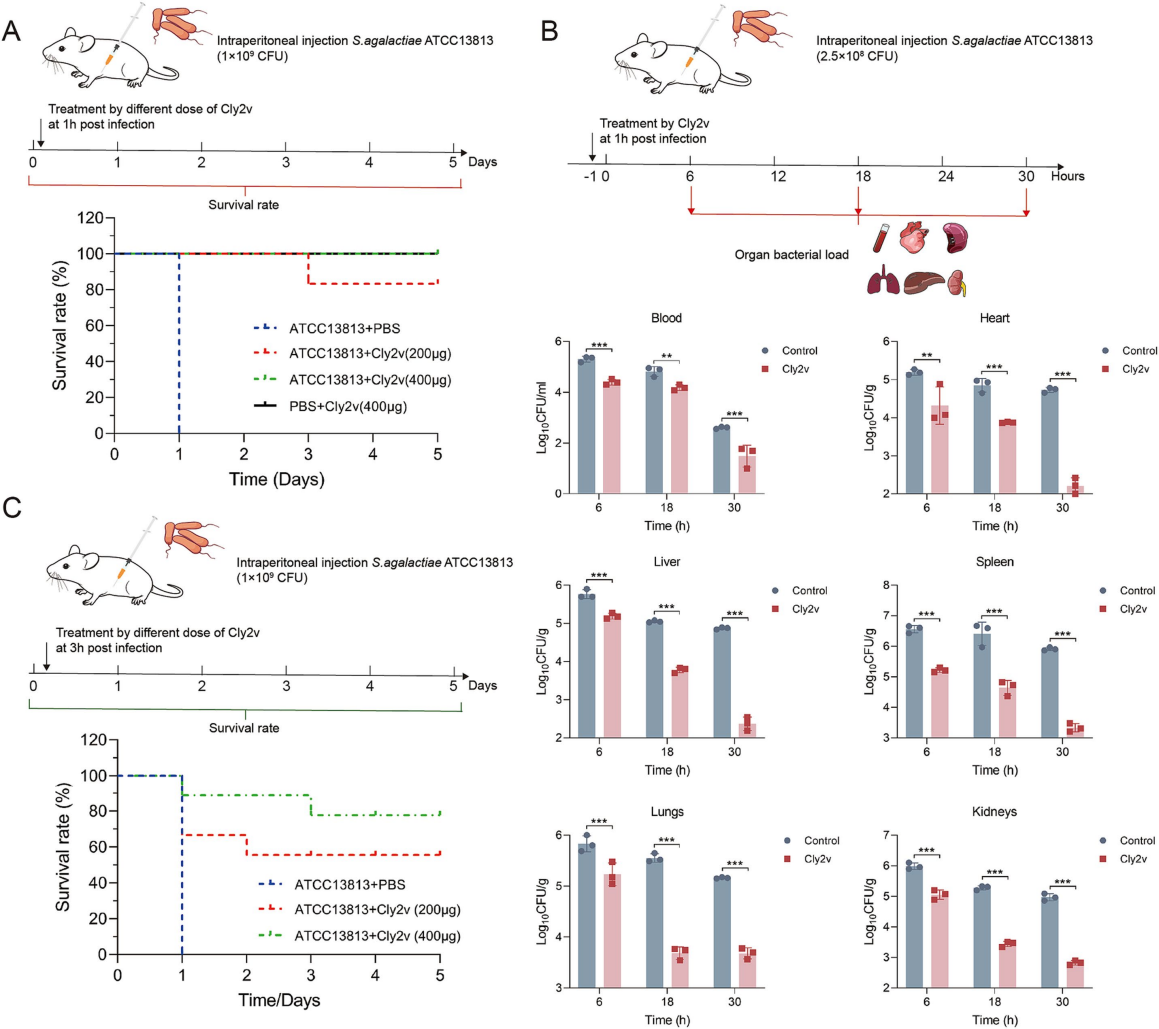
Therapeutic effects of Cly2v on systemic infection caused by *S. agalactiae* ATCC13813. **(A)** The survival rate of mice treated with 200 μg or 400 μg of Cly2v after 1 h of infection with 1 × 10^9^ CFU of *S. agalactiae* ATCC13813 (*n* = 6). **(B)** Bacterial load in blood, heart, liver, spleen, lungs, and kidneys of mice at 6 h, 18 h, and 30 h post-treatment with Cly2v. **(C)** Survival rate of mice challenged with 1 × 10^9^ CFU of *S. agalactiae* ATCC13813 after treatment with Cly2v (200 μg and 400 μg) at 3 h post-infection (*n* = 9). The experimental results are presented as the mean ± SD. Statistical significance was analyzed using two-way ANOVA (* *p* < 0.05; ** *p* < 0.01; *** *p* < 0.001).

To further evaluated the therapeutic efficacy of Cly2v against *S. agalactiae* infection *in vivo*, we established a systemic infection model in mice and treated with different doses of Cly2v. The results demonstrated that Cly2v exhibited significant protection for mice infected with *S. agalactiae* ATCC13813. Mice administered an intraperitoneal injection of 200 μg of Cly2v had a survival rate of 83% within 5 days, and those given a dose of 400 μg had a survival rate of 100%, whereas all mice in the control group died within 24 h (Figure 5A). Additionally, the bacterial load in the blood and various organs of mice was determined at different time points following treatment to further evaluate the therapeutic efficacy of the chimeric endolysin Cly2v. The results demonstrated a significant reduction in bacterial load in the blood and organs at 6 h, 18 h, and 30 h post-treatment (Figure 5B). Specifically, at 30 h post-treatment, the bacterial loads in the blood, heart, liver, spleen, lungs, and kidneys were 1.49 log_10_CFU/mL, 2.22 log_10_CFU/g, 2.37 log_10_CFU/g, 3.33 log_10_CFU/g, 3.68 log_10_CFU/g, and 2.82 log_10_CFU/g, representing decreases of 90.1, 99.7, 99.7, 99.7, 96.6, and 99.3%, respectively. Notably, treatment with 400 μg of Cly2v at 3 h post-infection provided a protection rate of 78% (Figure 5C). Although this was less effective compared to treatment at 1 h post-infection, Cly2v still demonstrated efficacy against late-stage sepsis induced by streptococcal infection.

## 4 Discussion

Bovine mastitis is regarded as a significant challenge in the dairy farming industry, threatening the advancement of livestock production and causing substantial economic losses (Rollin et al., 2015; Nyman et al., 2018). *Streptococcus*, including *S. uberis*, *S. agalactiae*, *and S. dysgalactiae*, are major pathogens responsible for bovine mastitis (Kabelitz et al., 2021). Although antibiotics are commonly used to combat bovine mastitis, the prevalence of multidrug-resistant bacteria and the risk of antibiotic residues in the food chain and dairy products highlight the urgent need to develop novel antibiotic alternatives.(Gomes and Henriques, 2016; Tomazi and dos Santos, 2020). Endolysins have been widely reported for their independent bactericidal activity *in vitro* and gathered significant attention from researchers (Fischetti, 2010; Linden et al., 2021).

Endolysins usually possess a classic modular structure which provide new directions for modification and design (Yang et al., 2014a). In this study, we constructed a novel chimeric endolysin, Cly2v, which was composed of the CHAP catalytic domain of Ply2741 and the CBD domain of PlyV12. *In vitro* experiments demonstrated that Cly2v exhibited stronger bactericidal activity against *S. uberis*, *S. agalactiae*, *and S. dysgalactiae* compared to the parental endolysin Ply2741 (Figure 2A). This adaptive modification could expand the application scenarios of endolysins, allowing for the development of more effective chimeric endolysins targeting different pathogens. Yang et al. demonstrated that the *S. suis* was more susceptible to chimeric endolysin ClyV rather than its parental endolysin GBS180 (Huang et al., 2020). Additionally, the chimeric endolysin ClyQ showed a broader lytic spectrum and delayed the development of mupirocin resistance in *S. aureus* compared to its parental endolysin LysGH15 (Duan et al., 2023). The CHAP domain usually plays a catalytic role on the bacterial peptidoglycan, while the CBD domain is able to binds to peptidoglycan sites on the bacterial cell wall (Gonzalez-Delgado et al., 2020; Zhou et al., 2020). The CBD domain is critical for the bactericidal spectrum and activity. Gu et al. demonstrated that LysGH15CBD can only bind to *S. aureus*, thus exhibiting lytic activity exclusively against this species (Gu et al., 2011) species. Conversely, the chimeric endolysin Ply187N-V12C, constructed by Dong et al., displays a broader lytic spectrum and enhanced activity because its V12CBD can bind to *Streptococcus*, *Staphylococcus*, and *Enterococcus* species (Dong et al., 2015). Compared to the parental endolysin Ply2741, Cly2v possesses the same CHAP domain. The enhanced activity of Cly2v may be attributed to the stronger binding affinity of CBD to *Streptococcus* causing bovine mastitis, thereby maximizing the catalytic function of the CHAP domain (Dong et al., 2015). These results imply that modifying and designing endolysins can significantly enrich the endolysin database and enhance their bactericidal activity and application prospects.

Bacteria typically form biofilms by extracellular polymer, which confer resistance to antibiotics and the host immune system (Berger et al., 2010). Biofilms can increase tolerance to antibiotics by up to 1,000-fold and serve as reservoirs for multidrug-resistant bacteria (Hall and Mah, 2017). Bacterial biofilms, along with somatic cells, can clog mammary ducts, making it difficult for antibiotics to penetrate the mammary tissue effectively (Gutiérrez et al., 2020). Remarkably, the chimeric endolysin Cly2v not only inhibited the formation of bacterial biofilms but also significantly removed mature biofilms. After treatment with Cly2v, bacterial biofilms became noticeably thinner, and the biomass significantly decreased (Figure 3E). Bacteria can resist antibiotic eradication by adapting to a biofilm lifestyle or by ceasing growth due to nutrient limitation (Ciofu et al., 2022). However, endolysins are independent of bacterial metabolic mechanisms by directly targeting bacterial peptidoglycans and penetrating deeper into the bacteria. Additionally, certain components in milk may affect the efficacy of endolysins against mastitis. Therefore, we investigated the bactericidal activity of Cly2v in milk. Compared to PBS buffer, the bactericidal activity of Cly2v was significantly reduced in milk. Previous studies have found that fat globules in milk could bind to host bacteria, leading to aggregation and bacterial clumping, which might affect the stability and bactericidal activity of endolysins in milk (O’Flaherty et al., 2005; Schmelcher et al., 2012).

Although Cly2v had shown high efficiency bactericidal activity against streptococci-induced mastitis *in vitro* and in whole milk, previous studies suggested that the *in vitro* bactericidal activity of antimicrobial agents such as endolysins does not always correlate with bactericidal activity within mammary tissues (Apparao et al., 2009; Demon et al., 2012). This phenomenon may be due to interactions between bacteria and host immune cells in the mammary tissue, which can lead to a decrease in drug efficacy in mammary gland (Demon et al., 2013). Therefore, it is crucial to establish suitable animal models to evaluate the therapeutic efficacy of drugs. Mouse mastitis models have been widely used for evaluating agents for mastitis (Sanchez et al., 1994). Previous studies on the clinical application of endolysins in bovine mastitis have primarily focused on *S. aureus* (Zdunczyk and Janowski, 2020). Only natural endolysins Ply0643 and Lys0859 have shown significant therapeutic effects against streptococci-induced bovine mastitis in murine mastitis models (Liu et al., 2022; Li et al., 2023). In a mouse model, Cly2v could significantly reduce the bacterial load and inflammatory cytokines levels in mouse mammary tissues, demonstrating significant therapeutic efficacy against early-stage streptococci-induced mouse mastitis (Figures 4B–E). Schmelcher et al. found that treatment with λSA2 (25 μg/gland) and B30 (250 μg/gland) reduced the number of *S. agalactiae* in the mouse mammary gland by 2.0 and 4.5 log at 45 min post-infection, respectively (Schmelcher et al., 2015). Similarly, the endolysin Ply0643 (100 μg/gland) effectively treated *S. agalactiae*-induced bovine mastitis, reducing bacterial counts in mammary tissue from 6.4 × 10^4^ CFU/g to 4 × 10^2^ CFU/g at 1 h post-infection (Liu et al., 2022). Although the number of bacteria decreased by Cly2v in the mammary gland less than B30, it can exert bactericidal activity at a lower dose, suggesting that Cly2v could be more cost-effective in future clinical applications.

Notably, treatment at 1 h post-infection may lead to insufficient colonization of bacteria in the mammary tissue. However, Schmelcher et al. found that the time interval between infection and treatment may not be critical, potentially due to the relatively low number of resident phagocytes in mouse mammary tissue, which leads to a faster infection rate compared to cows (Schmelcher et al., 2012). Guo et al. also demonstrated significant therapeutic efficacy with phage therapy at 6 h post-infection (Guo et al., 2024). Therefore, the effect of Cly2v in the treatment of late-stage bovine mastitis warrants further investigation. To our knowledge, Cly2v is the first chimeric endolysin evaluated in a mouse model of streptococci-induced mastitis, highlighting the potential of engineered endolysins for treating bovine mastitis and significantly enriching the endolysin database. Additionally, endolysins demonstrated significant efficacy in treating multidrug-resistant bacteria (Zhang et al., 2023), suggesting that Cly2v may also have advantages for treating MDR infections in bovine mastitis. Future research will focus on exploring the combination of endolysin Cly2v with antibiotics to assess their synergistic effects against bacterial infections.

*Streptococcus agalactiae* is not only a major pathogen of bovine mastitis but also causes serious diseases such as meningitis, neonatal pneumonia, and sepsis (Johri et al., 2006). Bacterial mastitis can further lead to severe systemic infections. Therefore, we established a systemic infection model with *S. agalactiae* to evaluate the therapeutic efficacy of Cly2v. Remarkably, Cly2v significantly improved the survival rate of mice infected with *S. agalactiae*, with a dose of 200 μg/mouse resulting in an 83% survival rate (Figure 5B). Furthermore, treatment with Cly2v led to a significant reduction in bacterial load in the blood and various organs. Interestingly, when treatment with 400ug of Cly2v at 3 h post-infection, the survival rate of the mice was only 78%. While Xi et al. observed that systemic infection occurred within 1 h after *Streptococcus* infection (Xi et al., 2023), delaying treatment until 3 h post-infection may result in irreversible damage to the mice tissues and organs, leading to a decreased survival rate. However, these results still demonstrated the significant efficacy of Cly2v in treating bacterial systemic infections. Importantly, Cly2v exhibited no toxicity to both the mammal cells and animal. These findings underscore the substantial potential of Cly2v as a novel antibiotic alternative.

In summary, we constructed a novel chimeric endolysin, Cly2v, which demonstrates more efficient bactericidal activity against *Streptococcus* causing bovine mastitis *in vitro* compared to the parental endolysin Ply2741. Additionally, in mouse models, Cly2v showed significant therapeutic effects against both early-stage mouse bovine mastitis and systemic infections induced by *S. agalactiae*. These results underscore the substantial potential of Cly2v in treating bacterial mastitis and provide new support and insights for further modification of endolysins.

## Data Availability

The datasets presented in this study can be found in online repositories. The names of the repository/repositories and accession number(s) can be found at: https://doi.org/10.6084/m9.figshare.27108784.v1.

## Footnotes

^1^ https://www.expasy.org/
