# Supplementary material for: A novel chimeric endolysin Cly2v shows potential in treating streptococci-induced bovine mastitis and systemic infections
Source: Front Microbiol. 2024 Oct 18;15:1482189. doi: 10.3389/fmicb.2024.1482189 (PMC11527626; doi:10.3389/fmicb.2024.1482189)
Supplement: Supplementary file 1 [file Data_Sheet_1.docx]

**Supplementary material**

**This file contains**

**1) Supplementary Figures S1 to S4**

**2) Supplementary Tables S1 to S3.**

**Supplementary Figure S1.**

**
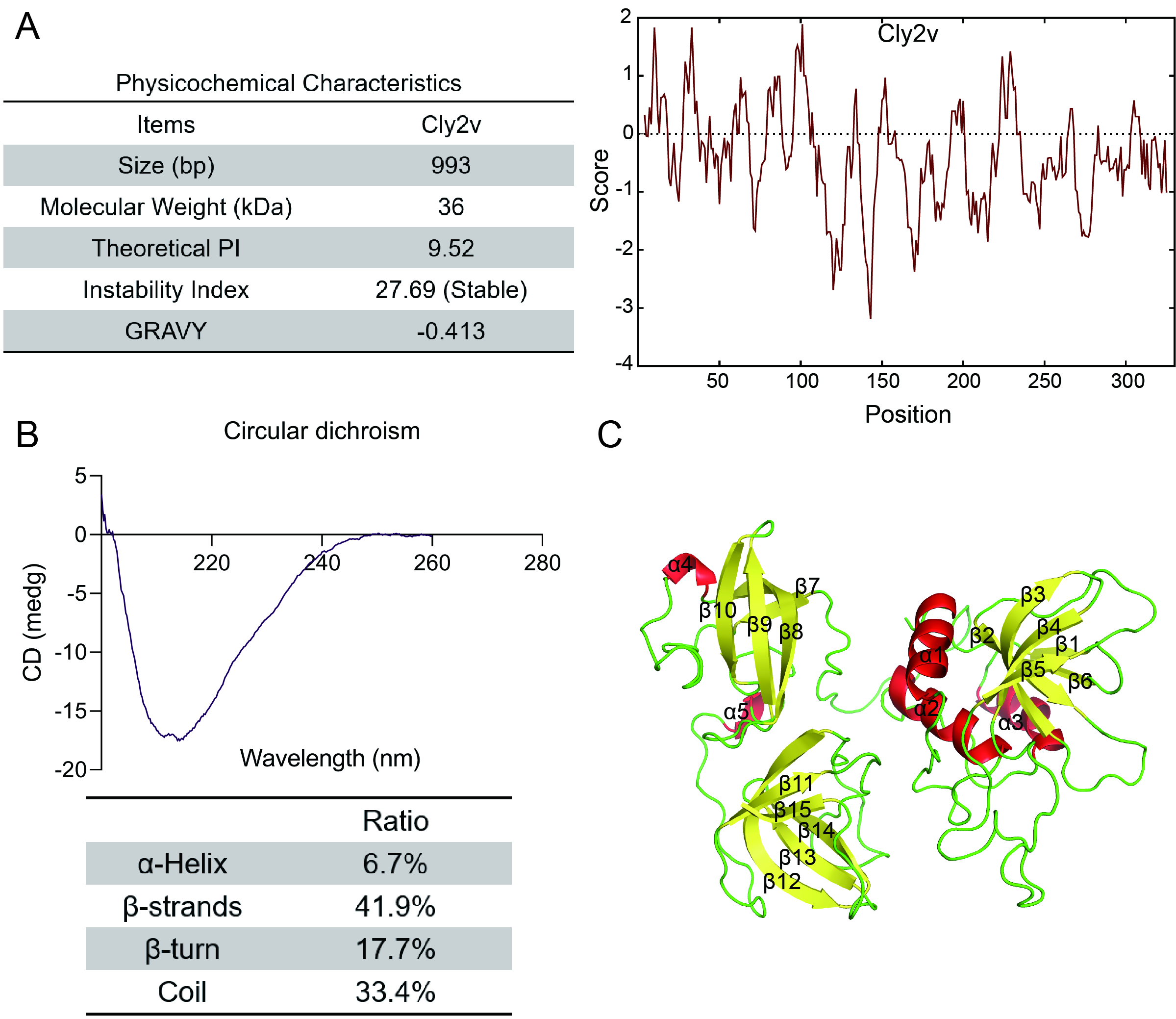
**

**Supplementary Figure S1.** Physicochemical properties and secondary structure analysis of Cly2v. **(A)** The physicochemical properties and hydrophobicity of the protein were analyzed using Expasy. **(B)** Circular dichroism results of Cly2v, displaying data specifically for the wavelength range of 200nm to 260nm. The detailed proportions of secondary structures are provided in the table. **(C)** Secondary structure of Cly2v, with α-helices, β-sheets, and coil structures indicated in red, yellow, and green, respectively.

**Supplementary Figure S2.**

**
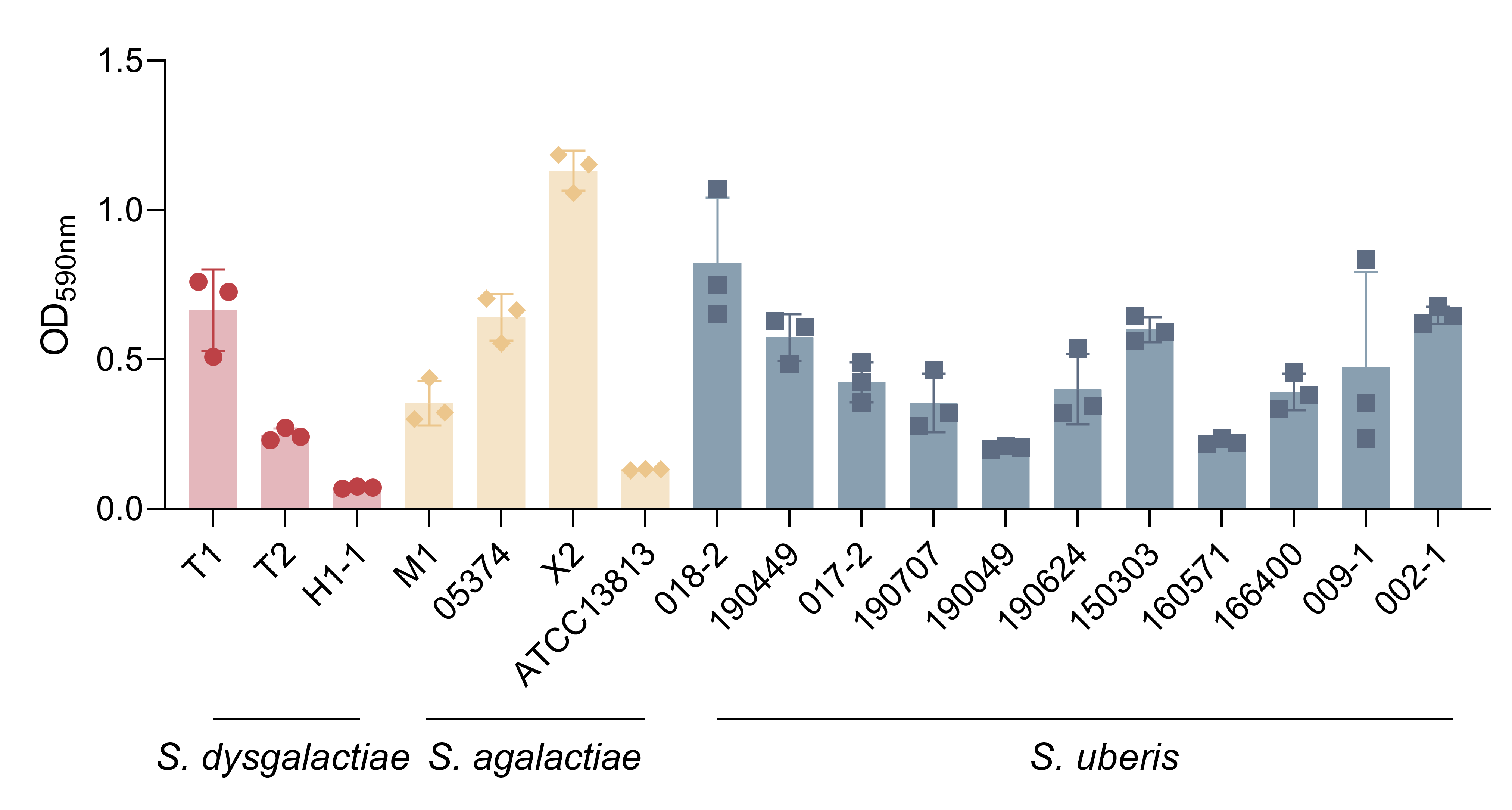
**

**Supplementary Figure S2.** Biofilm formation ability of *Streptococcus* species. The absorbance of OD_590nm_ represented the strength of the biofilm.

**Supplementary Figure S3.**

**
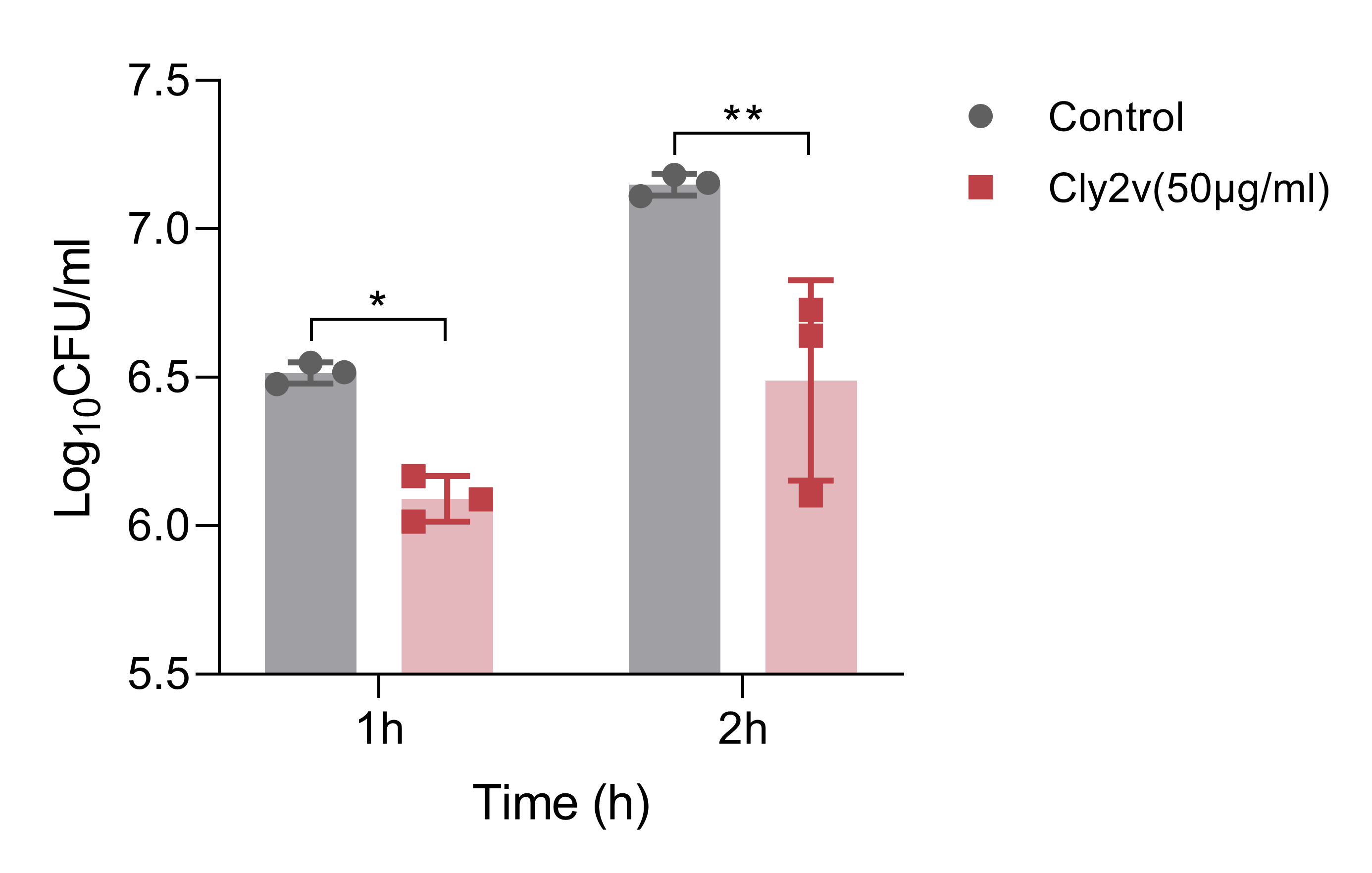
**

**Supplementary Figure S3. The effect of Cly2v to reduce *S. agalactiae* ATCC13813 in whole milk.** Milk was infected with 1×10^5^ CFU/ml of *S. agalactiae* ATCC13813 for 1 h and 2 h, respectively, and incubated with 50μg/ml Cly2v at 37°C for 1 h. The experimental results are presented as the mean of three independent experiments. Statistical significance was analyzed using two-way ANOVA (* *p*<0.05; ** *p*<0.01; *** *p*<0.001).

**Supplementary Figure S4.**

**
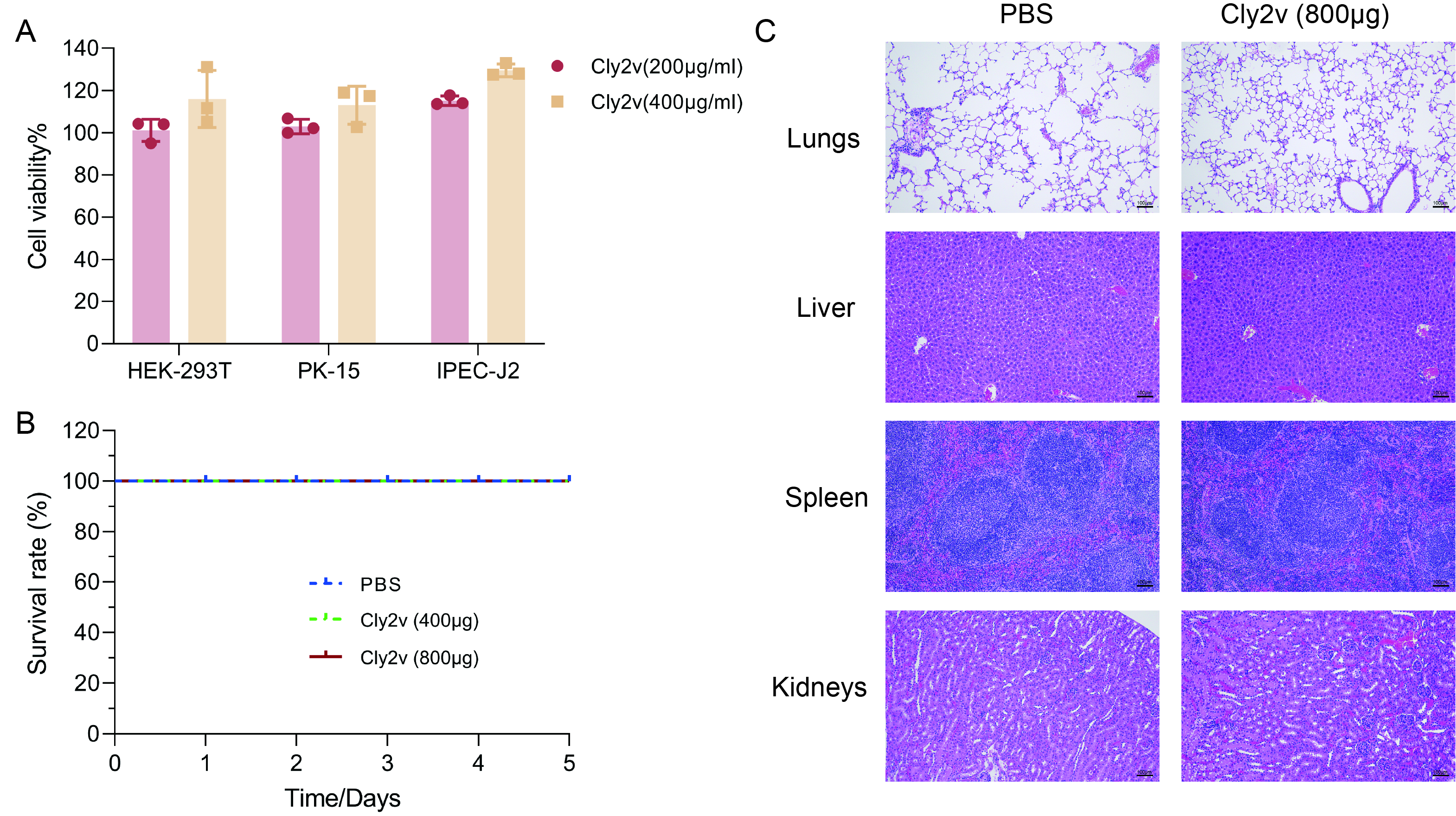
**

**Supplementary Figure S4. Toxicity evaluation of Cly2v on mammalian cells and mice.** **(A)** Toxicity evaluation of Cly2v on mammalian cells. Cell viability after Cly2v (200μg and 400μg) treatment was evaluated via CCK-8 assay. Results represent the means of three independent experiments, with statistical analysis performed using two-way ANOVA (*, *p*<0.05; **, *p*<0.01; ***, *p*<0.001). **(B)** Survival rates of mice receiving different doses (400μg and 800μg) of Cly2v (n=5). **(C)** Histopathology analysis of the liver, spleen, lungs, and kidneys of mice treated with PBS or 800μg of Cly2v at 5 d, with magnification ×200.

**Supplementary Table S1. All strains used in this study**

| **Species** | **Name** | **Source** |
| --- | --- | --- |
| *Streptococcus agalactiae* | X2 | Clinical isolate |
| *Streptococcus agalactiae* | M1 | Clinical isolate |
| *Streptococcus agalactiae* | ATCC13813 | Lab strain |
| *Streptococcus agalactiae* | 05374 | Clinical isolate |
| *Streptococcus dysgalactiae* | H1-1 | Clinical isolate |
| *Streptococcus dysgalactiae* | T1 | Clinical isolate |
| *Streptococcus dysgalactiae* | T2 | Clinical isolate |
| *Streptococcus dysgalactiae* | T3 | Clinical isolate |
| *Streptococcus uberis* | 018-2 | Clinical isolate |
| *Streptococcus uberis* | 190624 | Clinical isolate |
| *Streptococcus uberis* | 150303 | Clinical isolate |
| *Streptococcus uberis* | 190449 | Clinical isolate |
| *Streptococcus uberis* | 190049 | Clinical isolate |
| *Streptococcus uberis* | 166400 | Clinical isolate |
| *Streptococcus uberis* | 160571 | Clinical isolate |
| *Streptococcus uberis* | 190-1 | Clinical isolate |
| *Streptococcus uberis* | 190707 | Clinical isolate |
| *Streptococcus uberis* | 017-2 | Clinical isolate |
| *Streptococcus uberis* | 013-2-2 | Clinical isolate |
| *Streptococcus uberis* | H13-2-3 | Clinical isolate |
| *Streptococcus uberis* | 009-1 | Clinical isolate |
| *Streptococcus uberis* | 002-1 | Clinical isolate |

**Supplementary Table S2. All primers used in this study**

| **Name** | **Primers** | **Use** |
| --- | --- | --- |
| Ply2741-F | tttggatccatgacaacagcaaatg | *Construction of Cly2v plasmid* |
| Ply2741-R | cgatccagacgagcctcctaacctaaaccaacca |  |
| PlyV12CBD-F | ggaggctcgtctggatcgctaatgggggatcaaca |  |
| PlyV12CBD-F | tttaagcttttatttgaaagtacccca |  |
| RT-GAPDH-F | TGTTCCTACCCCCAATGTGT | *qRT-PCR assay* |
| RT-GAPDH-R | GGTCCTCAGTGTAGCCCAAG |  |
| RT-TNF-α-F | CCACGCTCTTCTGTCTACTG |  |
| RT-TNF-α-R | ACTTGGTGGTTTGCTACGA |  |
| RT-IL-6-F | GAGCCCACCAAGAACGATA |  |
| RT-IL-6-R | TTGTCACCAGCATCAGTCC |  |

**Supplementary Table S3. The survival rate of mice infected with different dose of *S. agalactiae* ATCC13813**

| **Dose** | **Survival rate (24h)** | **Survival rate (48h)** |
| --- | --- | --- |
| 4×10^9^CFU | 0% (0/6) | 0% (0/6) |
| 2×10^9^CFU | 0% (0/6) | 0% (0/6) |
| 1×10^9^CFU | 33% (2/6) | 0% (0/6) |
| 5×10^8^CFU | 67% (4/6) | 67% (4/6) |
| 2.5×10^8^CFU | 100% (6/6) | 100% (6/6) |
